# Neuropsychiatric Disorders and COVID-19: What We Know So Far

**DOI:** 10.3390/ph14090933

**Published:** 2021-09-17

**Authors:** Fernanda Majolo, Guilherme Liberato da Silva, Lucas Vieira, Cetin Anli, Luís Fernando Saraiva Macedo Timmers, Stefan Laufer, Márcia Inês Goettert

**Affiliations:** 1 Post-Graduate Program in Biotechnology, Universidade do Vale do Taquari-Univates, Lajeado 95914-014, Rio Grande do Sul, Brazil; fmajolo@univates.br (F.M.); cetin.anli@outlook.de (C.A.); luis.timmers@univates.br (L.F.S.M.T.); 2 Postgraduate Program in Medical Sciences Center, Universidade do Vale do Taquari-Univates, Lajeado 95914-014, Rio Grande do Sul, Brazil; gibaliberato@univates.br (G.L.d.S.); lucas.vieira@universo.univates.br (L.V.); 3 Department of Pharmaceutical and Medicinal Chemistry, Institute of Pharmacy, Eberhard Karls Universität Tübingen, 72076 Tübingen, Germany; stefan.laufer@uni-tuebingen.de; 4 Tübingen Center for Academic Drug Discovery (TüCAD2), 72076 Tübingen, Germany

**Keywords:** angiotensin-converting enzyme 2 (ACE2), central nervous system, SARS-CoV-2

## Abstract

SARS-CoV-2 (Severe Acute Respiratory Syndrome Coronavirus-2) affects the central nervous system (CNS), which is shown in a significant number of patients with neurological events. In this study, an updated literature review was carried out regarding neurological disorders in COVID-19. Neurological symptoms are more common in patients with severe infection according to their respiratory status and divided into three categories: (1) CNS manifestations; (2) cranial and peripheral nervous system manifestations; and (3) skeletal muscle injury manifestations. Patients with pre-existing cerebrovascular disease are at a higher risk of admission to the intensive care unit (ICU) and mortality. The neurological manifestations associated with COVID-19 are of great importance, but when life-threatening abnormal vital signs occur in severely ill COVID-19 patients, neurological problems are usually not considered. It is crucial to search for new treatments for brain damage, as well as for alternative therapies that recover the damaged brain and reduce the inflammatory response and its consequences for other organs. In addition, there is a need to diagnose these manifestations as early as possible to limit long-term consequences. Therefore, much research is needed to explain the involvement of SARS-CoV-2 causing these neurological symptoms because scientists know zero about it.

## 1. Introduction

The World Health Organization (WHO) classified coronavirus disease 2019 (COVID-19) as “a public health emergency of international concern” [1]. Similar to the virus that caused severe acute respiratory syndrome (SARS) in 2003, the new coronavirus was named as the severe acute respiratory syndrome coronavirus 2019 (SARS-CoV-2) [2]. A global pandemic in 2011, known as the Middle East Respiratory Syndrome (MERS), was caused by *Beta coronavirus*, which is from a zoonotic origin [3]. SARS-CoV-2 has affected 222 countries and territories with 179,696,026 confirmed cases and 3,891,297 confirmed deaths, as of 22 June 2021 (www.worldometers.info/coronavirus) (accessed on 22 June 2021). Considering the high mortality rate from infection caused by the SARS-CoV-2 and that the evidence on COVID-19 suggests that neurological events may occur in a significant number of patients [1], this manuscript aims to review and update data regarding neurological disorders in COVID-19.

## 2. Manifestations

Neurological manifestations in patients who are infected with the virus are mild or absent; however, everything is still quite new regarding COVID-19. Thus, we are learning to deal with the disease and understand its mechanism better over time. Currently, it is known that SARS-CoV-2 affects the central nervous system (CNS). As some of these manifestations can generate devastating sequelae, it is necessary to be attentive to the neurological effects in patients who are infected [2].

According to an updated review, neurological involvement is not uncommon and can result in serious complications unless detected and managed early. Ahmad and Rathore [4] pointed out that these complications were mostly observed in severely ill patients, and in some cases could even precede the respiratory symptoms [4].

Studies have shown that patients with COVID-19 who have an underlying neurological impairment have a greater vulnerability to more serious diseases. Patients with a pre-existing cerebrovascular disease may be at a higher risk of admission to the ICU and mortality. Moreover, according to Herman et al. [5], patients hospitalized with COVID-19 showed a 6–36% incidence of neurological events during their illness [5]. Therefore, COVID-19 may be strongly related to secondary neurological complications. During the pandemic, it is necessary to reassess consultations to attend neurological needs [5].

Vonck et al. [6] describe neurological manifestations in patients with COVID-19 and searched for the combination of COVID-19 and neurology terminologies up to May 10th, 2020. According to these authors, in the first large series of 214 patients with a laboratory-confirmed diagnosis of SARS-CoV-2, hospitalized in three COVID-19 hospitals in Wuhan, neurological manifestations were diagnosed in 36.4% of the patients [7]. Neurological symptoms were more common in patients with severe infections regarding their respiratory status (45.5% vs. 30.2% in non-severe cases): CNS manifestations (dizziness, headache, impaired consciousness, acute cerebrovascular disease, ataxia, and seizure), cranial and peripheral nervous system manifestations (impairment in taste, smell, and vision, and neuropathy), and skeletal muscle injury manifestations. Patients with severe respiratory infection were older, had more underlying disorders, and showed less typical symptoms such as fever and cough [6]. According to Mao et al. [7], to avoid losing the chance of treating and preventing further transmission, clinicians should suspect SARS-CoV-2 infections as a differential diagnosis, during the epidemic period of COVID-19, when seeing patients with neurologic manifestations. Furthermore, it would prevent delayed diagnoses or even misdiagnoses.

We can divide the manifestations of COVID-19 into two phases, namely, the initial stage, lasting approximately three to five days when the virus tries to replicate in the lungs and causes damage to the organs within some days [3]. With the appearance of clinical symptoms, the patient can also show signs of neurological symptoms. Concurrently, the complete blood and lymphocyte count should be monitored because lymphopenia is often found in hospitalized patients with COVID-19. If the patient shows improvement over the days, it is a sign that virus replication has been contained, mitigating the symptoms; otherwise, the patient enters the second stage, which is the severe infection stage. Here, virus replication in the lungs reaches an acute level of damage, with the appearance of fever, cough, and shortness of breath. The lung starts to show a typical glass appearance or changes into the type of acute respiratory distress syndrome (ARDS) [8]. From now on, the cytokine storm can lead to the failure of multiple organs, and thus the use of ventilatory support is likely. It is important to pay attention to opportunistic infections, such as staphylococci and pneumococci, which can progress to a serious infection [9].

In the short term, cerebrovascular events, headache, dizziness, encephalopathies, anosmia, ageusia, and mood problems are some of the neuropsychiatric complications [10]. The possibility of delayed neurological and neuropsychiatric complications caused by SARS-CoV-2 infection can be explained by the presence of latent CoV hosted in neural and immune cells [10]. At present, these long-term complications are unknown, but one can speculate from our understanding of the mechanisms of COVID-19 in the CNS and the evidence for the long-term neuropsychiatric effects of SARS-CoV-1 and MERS [10] (Table 1).

### 2.1. Neurological Disorder

#### 2.1.1. Meningoencephalitis

Helms et al. [11] reported neurological characteristics, which are mentioned above in an observational series in 58 of 64 consecutive patients admitted to the hospital in Strasbourg, France, because of ARDS due to COVID-19. Neurological findings were recorded in 14% of patients upon admission to the ICU (before treatment) and 67% when sedation and a neuromuscular blocker were withheld. Magnetic resonance imaging (MRI) of the brain was performed in 13 patients. Although these patients did not have focal signs that suggested a stroke, they underwent MRI because of unexplained encephalopathic features. In the eight patients who underwent electroencephalography, only nonspecific changes were detected. In general, ARDS due to SARS-CoV-2 infection was associated with encephalopathy, prominent agitation and confusion, and corticospinal tract signs. Two out of thirteen patients who underwent brain MRI had acute ischemic strokes [11].

Due to the cytokine flow, it is reasonable to assume that multiple severe inflammatory diseases can occur during a COVID-19 infection. This is also confirmed by clinical studies, which show that it is not uncommon for severe inflammatory reactions, such as encephalitis, to occur [12]. In a clinical study in a EUCOVID facility specifically made for patients suffering from encephalitis, it was shown that 32 of the 42 patients tested positive for SARS and that this could be a possible cause of the infection [12]. In addition, cases of acute disseminated encephalomyelitis (ADME) and myelitis, as well as meningitis have been reported [13,14,15].

#### 2.1.2. Anosmia and Ageusia

In a retrospective study [16], olfactory loss lasted, on average, 8–9 days. This represents an early symptom of a COVID-19 infection, which has been confirmed in more than 20 studies [17]. However, there are also cases in which patients were unable to smell for weeks and months afterward [18]. The reason for this is probably the direct destruction of nerve cells in the nose and the olfactory bulb [19].

Ageusia is, like anosmia, another common cranial nerve disorder that occurs in COVID-19.

#### 2.1.3. Acute Cerebral Vascular Disease

Individuals infected with COVID-19 may develop seizures because of hypoxia, metabolic disorders, organ failure, or brain damage. Through the detection of SARS-CoV-2 RNA in the cerebrospinal fluid (CSF), meningitis/encephalitis was reported to be associated with SARS-CoV-2 accompanied by seizures [20]. A patient affected by COVID-19 was reported with his primary presentation as focal status epilepticus [21,22].

Most patients suffer from general neurological symptoms, such as headaches. About 41% of patients complained of mild to moderate headaches in the frontal lobe, most of which lasted until the third day of illness [23].

#### 2.1.4. Intracerebral Hemorrhage (ICH) and Cerebral Venous Sinus Thrombosis

An increased risk of intracerebral hemorrhage occurs in patients severely associated with thrombocytopenia [24]. The impact of COVID-19 infection can lead to a systemic inflammatory response causing vascular endothelial cell injury, which predisposes the patient to intracerebral hemorrhage [25].

#### 2.1.5. Stroke

Stroke is one of the many consequences of a SARS-CoV-2 infection due to the thrombotic vascular events. Younger patients are most prevalent in stroke events; indeed, having a stroke with COVID-19 increases 7.6-fold when compared with influenza, for example [26]. The reason for its prevalence among younger patients remains unclear.

#### 2.1.6. Seizure

A report of meningitis/encephalitis accompanied by seizures shows that COVID has been linked with seizure [20]. Therefore, because of hypoxia, metabolic derangements, high levels of pro-inflammatory cytokines in the nervous system, organ failure, or even cerebral damage caused by COVID-19, the patients could develop seizures [22]. The role of cytokines in COVID-19-associated seizures remains unclear [25].

#### 2.1.7. Ataxia

Further details about ataxia and COVID-19 are needed; however, ataxia has been reported in one patient [7] in addition to myoclonus and cerebellar ataxia [27].

#### 2.1.8. Myelitis

COVID-19 can induce brain and spine demyelination and acute transverse myelitis, and there were reported pneumonia, seizures, bilateral lower limb weakness, urinary retention, and constipation in patients [28,29].

#### 2.1.9. Rhabdomyolysis

It was found that rhabdomyolysis can occur both initially and during the disease, and patients must be treated accordingly [30]. Besides general neurological symptoms, SARS-CoV-2 also damages sensory nerve pathways. Patients complain of loss of smell (5.1–70.2%), ageusia (5.6–54.2%), and visual disturbances (1.4–7.4%) [31].

### 2.2. Neuropsychiatric Disorder

In addition to the immunological causes, psychological features can be triggered through a COVID-19 infection. Many patients complained of depression (32.6%), anxiety (35.7%), sleep disturbances (41.9%), trauma (30%), or even psychosis (4%) [31]. In summary, clinical studies have shown a multitude of neurological and neuropsychiatric manifestations in SARS-CoV-2 patients [31,32,33].

Visual disturbances affecting the facial nerve occur less frequently but are another indication of aggressive destruction of neural pathways in the brain [34].

#### 2.2.1. Depression and Anxiety

Dizziness (up to 30%) and fatigue (up to 70%) are other common symptoms of SARS-CoV-2 patients [35]. Another interesting neurological symptom that should not be ignored is rhabdomyolysis (up to 3.5%) [36].

#### 2.2.2. Sleep and Stress Disorders

The sleep disturbances were reported more in the female sex, which increases during the COVID-19 infection but decreases with time after the recovery [37,38,39]; for example, insomnia has been improved three months after recovery [38,40]. Sami et al. [41] reported that sleep disturbances are independent of the severity of acute COVID-19; however, other works showed that the severity of acute COVID-19 is a predictor of those disturbances [42,43].

Moreover, post-traumatic stress disorder (PTSD) has been reported throughout the pandemic as an indirect consequence of stress and altered daily life [44,45]. However, the systemic inflammation process has been reported to be a pathophysiological mechanism in the PTSD condition [46,47]. More than 7% of patients present PTSD, and this number has been higher in patients surviving a critical illness by COVID-19 [44,48,49]. The severity of COVID-19 infection has been considered as a risk factor for PTSD, where ICU patients developed more PTSD than non-ICU patients [50,51]. Little is known about cytokine levels associated with PTSD [52].

#### 2.2.3. Addiction and Substance Abuse

The consequences of quarantine during the COVID-19 outbreak were several, such as acute stress disorders, anxiety, irritability, poor work performance, insomnia, post-traumatic stress disorders, high psychological distress, depressive symptoms, and economic problems [53,54]. Therefore, the search for an “exhaust valve” unleashes the consumption of addictive substances and becomes eminent [55]. The drug and/or alcohol disorders triggered by those negative conditions reached high-risk groups as well as the general population [56,57,58]. An increase in the use of benzodiazepines (BZDs) and alcohol has been observed because, after the lockdown, this practice persists in the population. It is worth mentioning that the use of illicit drugs has returned to pre-lockdown levels, leading to an elevated risk of developing comorbid psychiatric disorders [59].

### 2.3. Post-COVID Neurological Manifestations

During the pandemic, more and more people were observed to have health problems even after they had overcome a SARS-CoV-2 infection. Persistent post-COVID syndrome also referred to as long COVID, is a pathologic entity, which involves persistent physical, medical, and cognitive sequelae following COVID-19 [60]. Thus, despite negative PCR tests, a considerable number of patients suffered from post-viral symptoms, such as chronic fatigue, stress, fibromyalgia, and so on [61]. SARS-CoV-2 causes problems both in the acute phase and after the disease. A PET scan of the brain was able to differentiate with 100% accuracy between patients who had SARS-CoV-2 in the past and those who had not by showing visible changes in the brains of COVID-19 patients compared with a control group of healthy people [62]. The massive release of proinflammatory mediators, which is referred to in the literature as a cytokine storm [63], is considered to be responsible for the neuronal changes in the brain [63]. Due to the permanent disturbance of the brain stem, neurological complications such as sudden infant death syndrome (SIDS), dysautonomia, coma, delirium, or even brain death can occur [64]. Studies in which brain autopsy was performed support the hypothesis that SARS-CoV-2 infiltrates and destroys the brain, as RNA of SARS was found in 30–40% of the brains [65].

#### 2.3.1. Parkinson’s and Alzheimer’s Disorders, Guillain–Barré Syndrome

Various neuronal abnormalities such as disturbance of sensory peripheral perception and pain of the nerves are among other manifestations that SARS can cause. The spectrum of neuropathies is broad and strongly overlaps with neuropathological features that have also been demonstrated in clinical studies. Neuropathies are particularly common in severe cases [66]. Damage to peripheral nerves in the form of Miller–Fisher syndrome occurs frequently as a result of the cytokine storm and as antibody-mediated neuropathy [67].

More serious, however, is the fact that SARS also accelerates or aggravates existing neuropathological diseases. For example, SARS-CoV-2 can lead to demyelination of the nerves in the brain and spine [68]. Both demyelination and SARS-CoV particles were found in some studies that performed autopsies of brains [69,70]. Diseases such as Alzheimer’s, Parkinson’s, Multiple Sclerosis (MS), and Guillain–Barre Syndrome are further advanced by delayed CNS damage after COVID disease, greatly increasing patients’ mortality [71].

#### 2.3.2. COVID-19 Infection and Loss of Memory

Memory is defined by the capacity to store and retrieve information [72]. Budsen et al. [73] describe four basic systems that are important for memory. The episodic, semantic, procedural, and working memory. Detailed knowledge of the anatomical structures in each system helps to draw a possible link between damaged areas in the brain due to SARS-CoV-2 and memory loss occurring during and after a COVID-19 disease. The following structures are necessary for memory. The temporal lobes of the hippocampus are involved in semantic and episodic memory. Furthermore, the thalamic nucleus, the fornix, and the prefrontal cortex are involved in the storage of information within minutes to years. The procedural memory in areas of the basal ganglia, cerebellum, and supplementary motor are primarily responsible for the ability to perform automated processes such as driving. The prefrontal cortex contains the working memory, which stores information within the last few seconds to minutes. Alteration in these structures leads to memory impairment [73].

Presently, clinical observations show that COVID-19 patients also complain of memory problems as one of many neurological manifestations. In other words, even if the virus can no longer be detected in the body, changes still take place. For example, 50% to 80% of those who had COVID-19 continue to experience symptoms three months after the onset of COVID-19 [74,75].

During the SARS-CoV-2 pandemic, persistent symptoms such as fatigue, dyspnea, hyposmia/anosmia, dysgeusia/ageusia, memory/cognitive impairment, sleeping alterations, and painful syndromes have clinically manifested as long COVID after a COVID-19 recovery [76]. With the help of ^18^F-FDG PET images, two clinical cases with a confirmed diagnosis of SARS-CoV-2 could be described quite early, in which hypometabolism occurred in the above-mentioned structures, which are crucial for our memory, in the brain [77]. Further findings were obtained in other long COVID patients. For example, 49% of 35 patients complained of memory/cognitive problems three weeks after the infection [76]. In addition, further images show cerebellar hypometabolism, which is also associated with memory/cognitive complaints [78,79]. The most common reason for this is probably the inflammation of the brain by the neurotropism of SARS-CoV-2 [80,81].

MRI images of COVID-19 patients show a statistically higher bilateral grey matter volume (GMV) in various brain areas, including the hippocampi. This shows a correlation with memory loss [82]. Global GMV, GMVs in left Rolandic operculum, right cingulate, bilateral hippocampi, and left Heschl’s gyrus were also found to correlate with memory loss (*p* value < 0.05). A total of 8 out of 61 patients show problems with memory, which is also clinically reflected in the above findings [82]. Likewise, atrophic changes in the hippocampus play a key role in memory loss as well as in Alzheimer’s disease. Such changes can be seen in a reported review of Moriguchi et al. [20]. The fact that COVID-19 can affect Alzheimer’s was already mentioned in the previous lines. SARS has been shown to have a general neuropsychiatric effect in 2004 and 2005 [83].

Significantly more problems with memory in COVID-19-positive patients after 30 and even after 60 days are seen, compared with a control group, which also shows that there must be a connection between the infection and the loss of memory function [84]. However, the authors point to a small number of patients, which means that the significance of this study should be treated with some caution [84]. Nevertheless, neurological symptoms such as memory loss, anosmia, and ageusia were significantly increased 30 days after infection, thus appearing to be COVID-19-associated long-term symptoms [84].

The loss of memory function is also shown in a study by Garrigues et al. [85]. Here, 120 patients were questioned about their condition for approximately 110 days after infection via a short phone questionnaire; 34% complained about memory loss and 28% about concentration problems [85]. In a Norwegian study of 13,001 patients, 11% of the cohort who tested positive for SARS-CoV-2 complained about memory problems. By contrast, only 2% of the control group complained about difficulties with memory. A strong association of COVID-19 and memory problems can be seen here (odds ratio [OR], 4.66; 95% CI, 3.25–6.66) [86]. Similar data are presented by Davis et al. [87] who evaluated cognitive and memory disbalance in 3203 patients. Of the examined patients, 55.9% reported memory problems within the first few weeks and 50.5% reported problems after sixth months from the onset of infection. Interestingly, no abnormal changes in the brain could be detected by MRI in 87% of patients [87].

There is no clear explanation for the symptoms thus far. However, within a few hours of infection, an infiltration of various inflammatory cells occurs, which probably leads to hypomyelination of the axons, which has been shown in experimental studies on animals and refers to cognitive dysfunction [88,89,90]. Prof. Harald Prüß, Director of the German Center for Neurodegenerative Diseases, assumes an antibody-mediated pathology that leads to neurogenesis [91]. Ultimately, memory loss is a multifactorial issue that must be further investigated.

One potential substance that could help counteract memory loss in the future is ghrelin. Ghrelin enters the hippocampus and binds to neurons there. By changing the formation of the synapses, learning processes and memory are induced [92]. To date, no connection between a COVID-19 and ghrelin could be established. Because of its endogenous function in the brain and the previous knowledge that ghrelin plays a role in inflammatory processes and cytokine flow, it remains a potential therapeutic approach in the treatment of neurological problems during a COVID infection [93,94]. Ghrelin has been shown to play a role in the treatment of neurological problems during a COVID-19 infection.

## 3. Mechanism

The SARS-CoV-2 is a single-stranded RNA enveloped by a sequence of amino acids [95]. Its RNA genome is 29903 nucleotides in size, encoding 9860 amino acids (www.ncbi.nlm.nih.gov/nuccore/1798174251) (accessed on 9 September 2021). As the RNA sequences between SARS-CoV-2 and SARS-CoV are 79.5% similar [3], their mechanisms of infections may also resemble each other [96]. It has previously been suggested that the angiotensin-converting enzyme 2 (ACE2), the receptor for cell adhesion, is a target of SARS-CoV infection, using the serine protease TMPRSS2 [96]. SARS-CoV-2 sheds nucleic acid from the host to spread, similar to the influenza virus, but its pattern of detachment is different from SARS-CoV [3]. It possesses more pathogenicity and transmissibility than SARS-CoV and MERS-CoV because SARS-CoV-2 and ACE2 have a stronger binding capacity [96]. Both epithelial and endothelial cells with the presence of the ACE2 receptor are present throughout the thorax and abdomen [97]. In the brain, its expression is considered to be lower; however, autopsy studies have previously demonstrated the presence of SARS-CoV particles in the brain tissue [98]. In addition, neurological deposition has been demonstrated in other beta-coronaviruses, including SARS-CoV, MERS-CoV, HCoV-229E, mouse hepatitis virus, and porcine hemagglutinating encephalomyelitis coronavirus (HEV) [99].

It is important to note that an infiltration pathway into the CNS may not be via ACE2 epithelial or endothelial cells, but rather through the trans-synaptic viral transfer after initial peripheral nerve invasion [96]. This explains the presence of the predominant virus in neurons [98]. There is also the possibility of a glial presence of ACE2 due to a higher relative prevalence of SARS-CoV in the brain (glial cells, which are most common) compared with the cerebellum [99].

**Figure 1:**
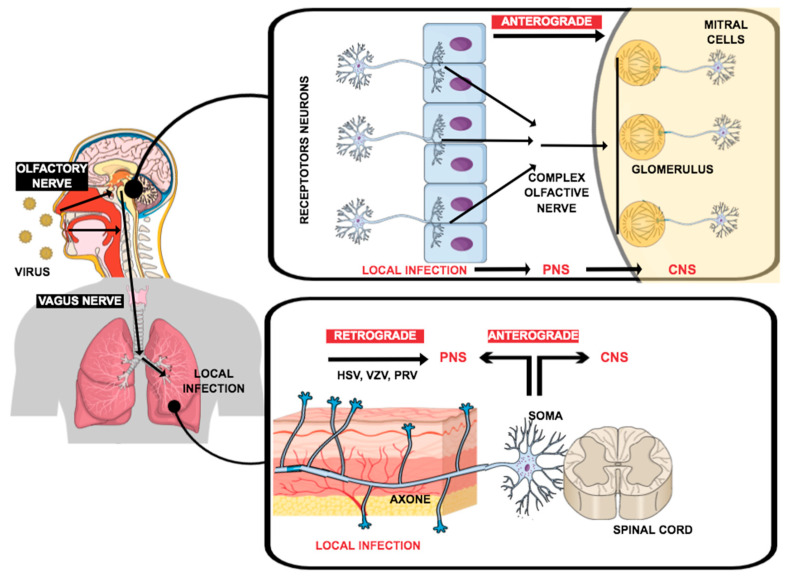
Pathways through which viruses can infect the peripheral nervous system (PNS) or central nervous system (CNS): (1) nerve endings were accessed in the tissues by the infection using axonal transport machinery to gain access to the CNS; or (2) by the infected cells in a circulatory system, which carries the infection through the blood–brain barrier into the CNS. Adapted from Yachou et al., 2020 [98].

There are several approaches in the literature that describe the route of infection of the virus into the nervous system. Finally, there are infections of both the central and peripheral nervous systems. As shown in Figure 1, SARS-CoV-2 enters the nerve pathways through the epithelial cells of the nose or pharynx. From now on, there are several possibilities to explain the transport into the respective nervous systems. In the first one, the anterograde pathway, the virus enters the CNS via axonal transport by infected epithelial cells in the PNS, from where it can permeate the blood–brain barrier and enter the CNS [100]. The other pathway is called the retrograde one. Here, the virus uses neuronal routes to enter the brain via retrograde transsynaptic propagation [99,101].

**Figure 2:**
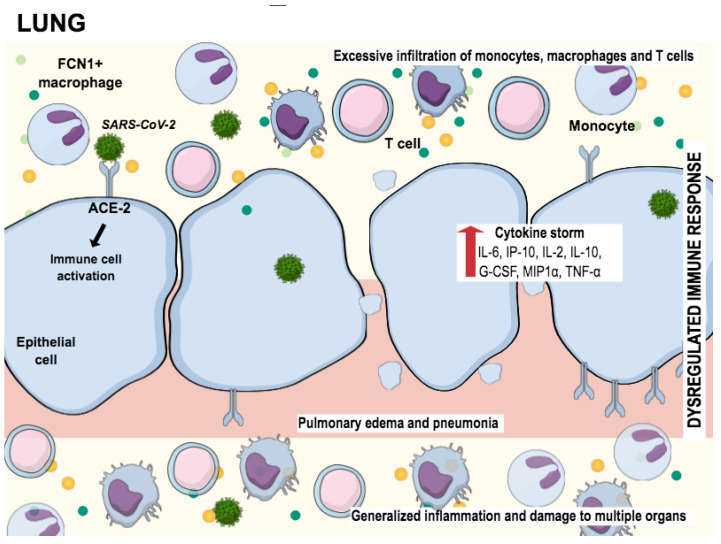
SARS-CoV-2 infecting cells in lung tissue through the interaction with angiotensin-converting enzyme 2 (ACE2), promoting the generation of pro-inflammatory cytokines (cytokine storm). This process attracts “defense” cells such as macrophages, monocytes, and T cells to the site of infection, triggering the inflammation process. The result of this inflammation is lung tissue damage. Adapted from Tay et al., 2020 [102].

In addition to the destruction of neuronal cells, SARS-CoV-2 massively attacks the lung cells. Figure 2 shows the way how the epithelial lung cells are damaged. After the virus has entered the epithelial cells through receptors such as ACE-2, a cascade of inflammatory reactions, dysregulations, and an excessive immune response occur. While releasing the virus out of the host cell, macrophages are triggered to activate cytokines and other chemokines. This reaction leads to massive further infiltration of other pro-inflammatory mediators, what we call a cytokine storm, which ends up in multiorgan damage [102].

**Figure 3:**
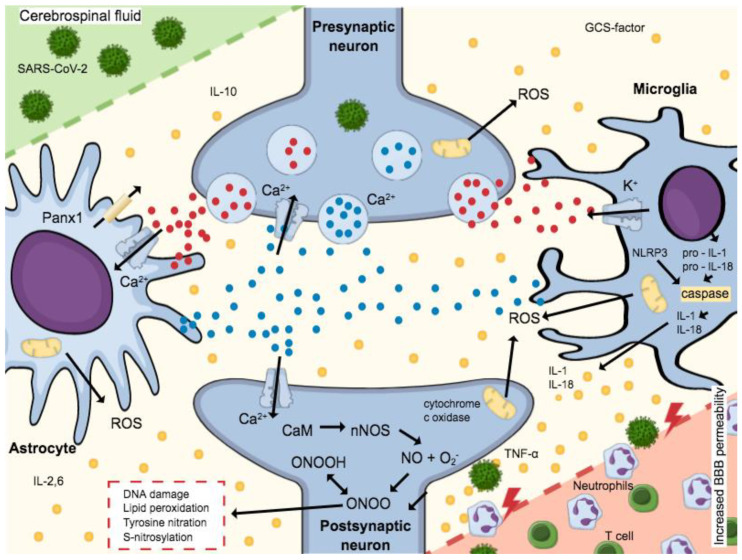
Microglia and astrocytes are activated by ATP expressed during the release of pro-inflammatory cytokines. NMDA receptors expressed by Glutamate activation allow the Ca^2+^-dependent exocytosis of ATP, thus releasing more Glutamate. A massive release of these neurotransmitters increases cell death and excitotoxicity, since postsynaptic neuron increased the Ca^2+^-calmodulin (CaM) complex formation and consequent nNOS activation. Neurotoxicity is mediated by NO production, which interacts with the iron–sulfur centers in the mitochondrial electron transport chain, and produces reactive oxygen species (ROS), impairing cellular energy production. Still, a reaction of O_2_^−^ (superoxide ion) and NO forms peroxynitrite (ONOO^−^) and peroxynitrous acid (ONOOH). Such formation leads to oxidative stress, which includes DNA damage, lipid peroxidation, tyrosine nitration, and excess S-nitrosylation, causing neuronal impairment and/or death. The activation of caspase-1 is mediated by the NLRP3 inflammasome by the cleavage of pro-IL-1β and pro-IL-18 in IL-1β and IL-18, respectively. The mature forms of cytokines are secreted worsening the neuroinflammatory process established. Then, the neuroinflammatory process established can be worsened by these mature cytokines. Adapted from Ribeiro et al., 2021 [105].

To date, clinical studies have demonstrated that SARS-CoV-2 causes neurological symptoms. It is assumed that the virus can cross the blood–brain barrier by a thus far unknown mechanism. According to the literature, SARS-CoV-2 leads to an increase in the permeability of the blood–brain barrier and thus facilitates the penetration of viral material. Figure 3 shows the complex biochemical mechanism by which this permeability reduction occurs. The basis for the destruction of cells and thus the compact structure of the tight junctions is the release of cytokines [103]. In addition to the cytokine storm, reactive oxygen species, which are formed after virus contact, are also responsible for the facilitated penetration. This mechanism seems logical, since comorbidities, such as hypertension, which increase mortality, also disrupt the blood–brain barrier [104].

### Inflammatory Markers on Neuro-COVID Pathogenesis

In COVID-19 patients, several inflammatory markers are associated with neuropathogenesis. This immunologic phenomenon is called “cytokine storm”, characterized by the increase in pro-inflammatory cytokine levels leading to a systematic inflammation, exacerbating viral pathogenesis causing sepsis, ARDS, and multiorgan failure [106,107,108,109,110]. Such molecules signalize various immune cells such as white blood cells (B cells) or T cells to attack the virus. However, cytokine storm could continue attacking the tissue even with no more presence of virus, thus considering the hyperinflammatory state [111].

The cytokines associated with COVID-19 hyperinflammatory process are IL-1β, IL-2, IL-4, IL-6, IL-7, IL-8, IL-9, IL-10, IL-18, IP-10, IFN-γ, and TNF-α, monocyte chemoattractant protein (MCP)-1, granulocyte stimulating factor (G-CSF), MCP-3, macrophage inflammatory protein 1α (MIP-1A), and cutaneous T-cell attracting chemokine (CTACK) [8]. IL-6, IL-1, and MCP-1 increase significantly in patients’ episodic tension-type headaches [112]. The IL-1b and IL-6 are two of the highest cytokines expressed during viral-like activation in the vulnerable brain regions [111]. Such cytokines are considered the key mediators in cytokine storm development due to inducing B cell proliferation, which promotes CD4+T cell response [113,114,115]. Their blockade could avoid deleterious effects due to the hyperexpression cytokine and protecting brain cells [116,117], suggesting that the use of IL6 antibodies in COVID-19 cases blocks the action against several tissues [111]. It is worth mentioning that, in severe cases of COVID-19 infections, the T cell counts (CD4+ and CD8+) were significantly lower than moderate infections. This kind of result suggests a progressive T cell exhaustion promoted by cytokine storm [25,118]. The hyperinflammation and systematic infection process by SARS-CoV-2 can lead to central nervous system (CNS) and peripheral nervous system (PNS) manifestations.

Approximately 5% of COVID-19 patients develop stroke, mostly ischemic, during hospitalization [7]. In stroke patients with COVID-19, the cytokines IL-1β, IL-6, and TNF-α are representative as well as in the CSF and blood serum [119,120,121,122]. Additionally, SARS-CoV-2 can induce encephalitis, with symptoms including altered consciousness, moderate nuchal rigidity, and akinetic syndrome with mutism, and showed elevated IL-6, IL-8, IFN-γ, and TNF-α during mainly the akinetic mutism [12,123,124]. IFN-γ was also observed to be elevated during viral meningitis [125]. Due to the virus contact with gustatory or olfactory, the COVID-19 patients showed cranial nerve symptoms such as hyposmia and dysgeusia, which might be caused by elevated IL-6 in saliva, plasma, and nasal mucus [126,127]. Moreover, SARS-CoV-2 has been associated with seizures, convulsions, and epilepsy [128,129,130], where high levels of IL-6, IL-1β, and TNF-α were observed after acute febrile convulsions. IL-6 elevations have been associated with tonic-clonic seizures [131].

Therefore, the cytokine storm triggered by SARS-CoV-2 has been associated with many neurological manifestations. Consequently, reduction in the viral load becomes an important factor for treatment or prophylaxis [25]. Suppressors of cytokine storm as well as anti-inflammatory drugs must be investigated as promising treatments for COVID-19, including stem cell therapy, TNF blockers, immunosuppressants, and IL-1 antagonists [25,121,122].

## 4. Pharmacological Approaches

Currently, research is ongoing for possible therapeutic approaches to help control COVID-19. Since search has thus far been rather modest, except for the already approved vaccines, it will be necessary to gain further information about potential targets within the complex pathomechanism of the COVID-19 disease, including biotechnological experiments, to make targeted treatment possible. Current treatments range from anticoagulants, steroids, ACE2 antagonists, and monoclonal antibodies to multidrug therapies. However, there is still a lack of drugs. Kaushik et al. [132] describe a new approach to reduce neurological complications by inhibiting TLR4, which plays a role in the immune response. Using the potential of phytochemicals that have been shown to have anti-inflammatory and antiviral properties is also considered to be effective against SARS [133].

## 5. Modelling the Disease

Developing an experimental model that mimics infection by COVID-19 is essential for enabling discoveries related to its pathogenicity; it is also necessary to find the interactions with the different hosts, thus establishing the criteria for the prevention and care of diseases developed in non-human primate models (Rhesus macaques), similar to SARS-CoV-2 [134]. Fulfilling Koch’s postulates, this animal model confirmed the causal relationship between respiratory disease in this nonhuman primate and SARS-CoV-2, documenting asymptomatic animals during the study, which was also observed in humans [135].

Bao et al. [135] utilized transgenic mice provided with ACE2 and considered the human cell entry receptor of SARS-CoV-2 to confirm their pathogenicity. Weight loss and virus replication were observed in the lung of mice infected with SARS-CoV-2. Additionally, interstitial pneumonia with infiltration of significant macrophages and lymphocytes into the alveolar interstitium and accumulation of macrophages in alveolar cavities were confirmed. The same injuries were not found in wild-type mice (without ACE2) during SARS-CoV-2 infection. The model enables detailed studies of the pathogenesis of this illness, which may play a critical role in the evaluation of therapeutic drugs and vaccines [135].

A 3D brain model with pluripotent stem cells differentiated into neurons and glial cells can be used to find the mechanisms behind the COVID infection. The so-called Brain Sphere models are infected with SARS. PCR is used to identify important proteins that SARS uses to invade the brain. ACE2, neuropilin-1, and TMPRSS2 are used by SARS-CoV-2 for this purpose. In addition, a high number of virus particles were found in neuronal cells suggesting that viral replication takes place in infected cells [7]. In another study, SARS-CoV-2 was shown to infect TUJ1 and nestin cells in 3D human brain organoids and to directly address neurons and neuronal progenitor cells (NPCs) [136].

There are also modeling experiments with human-induced pluripotent stem cells (hiPSC) that are differentiated into hNPCS cells, which were infected with SARS. It was observed that MAP2 and SOX2 cells tested positive and TUNEL cells tested mostly negative with SARS. Furthermore, a correlation between cell death and SARS was observed [137]. In addition, it has been confirmed that cells infected with SARS show enormous hyperbolic activity and at the same time an oxygen deficiency [137].

The use of cell cultivation models to identify potential antivirals is another important issue. The model of Ju et al. [138], in which an HTS (high throughput screening) of over 377 compounds was carried out, shows that potential antivirals can be identified in this way. Salinomycin, tubeimoside I, monensin sodium, lysine chloride, and nigericin sodium turned out to be promising. Further investigation with Caco-2 cell models and potential antivirals is therefore necessary.

## 6. Concluding Remarks

The clinical studies registered in ClinicalTrials.gov regarding neurological disorders and COVID-19 can be divided into the following groups: multiple sclerosis (NCT04354519, NCT04379661, NCT04355611, NCT04390126), encephalitis (NCT04361344, NCT04320472, NCT04359914), stroke (NCT04373109, NCT04377425, NCT04370197), dementia (NCT04385797, NCT04362930), and myositis (NCT04367350). Due to the immunological conditions of multiple sclerosis patients, COVID-19 might present a threat to them. Still, the risk estimate for multiple sclerosis patients remains unknown [139]. It is recommended that the clinicians instruct their patients about the potential risk of COVID-19 and follow the preventative measures for this disease.

In general, dementia studies aim to describe the neurological and psychiatric manifestations occurring in COVID-19 patients, as well as exploring the impact of social isolation. Surveys to document neurologic episodes and define the neurological profile in patients with COVID-19 were conducted in the USA and the Philippines. In this study, registering myositis, the researchers are verifying a multimodal assessment of neuromuscular pathology associated with SARS-CoV-2 infection partially resulting in elevated levels of creatine kinase in the hyperacute phase (NCT04386083 and NCT04379089).

The pandemic is not only important because of the high rates of lethality and dispersion, but also because of its possible evolution in the medium or long term. Therefore, in the future, the nervous system may have consequences of fundamental importance for infected individuals. As noted by Serrano-Castro et al. [80], SARS-CoV-2 causes a cytokine storm, which can have acute and unpredictable as well as long-term effects on the CNS. The pathogenic mechanisms of different neurological diseases have a neuroinflammatory basis that involves the factors stimulated in the third phase of the acute COVID-19 disease. Consequently, the progression of diseases in their neurodegenerative phase, such as Alzheimer’s, Parkinson’s, and multiple sclerosis, may be influenced. The monitoring of these patients is essential since neuroinflammatory dysregulation can be persistent in cases of severe diseases, following the model called inflammation-immunosuppression syndrome and persistent catabolism [80].

According to Matías-Guiu et al. [140], there will possibly be few changes in the medium-term future, primarily being related to prevention habits and the circulation of people, reducing the presence of numerous people in waiting rooms. Telemedicine and teaching virtualization could be new resources for broad use. However, the changes in the clinical examination or the indications for complementary examinations have not yet been generated, at least by the neurology experts [140].

The actual involvement of the neurological manifestations of COVID-19 is not yet clear, but it seems that individuals with severe illnesses have CNS involvement and neurological manifestations. Asadi-Pooya and Simani [141] listed different measures to be taken to clarify the roles played by this virus in the cause of neurological manifestations. Among them was to pay attention to the recording of neurological symptoms and signs, including changes in mental status; detailed clinical, neurological, and electrophysiological investigations of the patients; attempts to isolate SARS-CoV-2 from the CSF; and autopsies of the victims of COVID-19 [141].

From this still unknown scenario on the impact of COVID-19 on patients with neurological disorders, in addition to the highly recommended preventive measures, there is a need for the development of new neuroinflammatory drugs. Reversing and/or mitigating neurological damage has much to gain from the vast amount of data made available through scientific evidence, including epidemiological and clinical aspects of the disease, as well as genomic sequencing, biochemical characterization, enzymology, and resolution of protein crystallographic structures’ constituents of the virus. Prospective clinical studies should be encouraged to recognize the pathophysiological mechanisms and different forms of treatment for patients with these disorders.

## Figures and Tables

**Table 1:** Main neuropsychiatric disorders related to COVID-19, with its most common phase of presentation (during or after the infection and already before the infection or after).

Disorders	Disorders Presentation			
	Acute Phase during Infection	After Infection (3, 6, or 12 Months after)	Are Already Present before the Infection	Come after Infection
**Neurological Disorders**				
Headache and Dizziness	X	X		X
Anosmia and Ageusia	X	X		X
Acute ischemic stroke	X	X		X
Intracerebral hemorrhage (ICH)		X		X
Cerebral venous sinus thrombosis	X	X		X
Encephalopathy with symptoms that may range from mere headache, fever and neck rigidity	X	X		X
Seizure	X	X	X	
Ataxia		X		X
Myelitis		X		X
Rhabdomyolysis		X		X
Guillain–Barré syndrome		X		X
Stroke	X	X		
Alzheimer’s disease		X		X
Parkinson’s disease		X		X
Loss of memory		X		X
**Neuropsychiatric Disorders**				
Depression		X		X
Anxiety	X	X		X
Sleep disorders	X	X		X
Stress disorders		X		X
Addiction and substance abuse		X		X

## Data Availability

Data sharing not applicable.
